# Local Anesthetic-Induced Neurotoxicity

**DOI:** 10.3390/ijms17030339

**Published:** 2016-03-04

**Authors:** Mark Verlinde, Markus W. Hollmann, Markus F. Stevens, Henning Hermanns, Robert Werdehausen, Philipp Lirk

**Affiliations:** 1 Department of Anesthesiology, Academic Medical Center, University of Amsterdam, Meibergdreef 9, Amsterdam 1105AZ, The Netherlands; m.h.verlinde@amc.uva.nl (M.V.); m.w.hollmann@amc.uva.nl (M.W.H.); m.f.stevens@amc.uva.nl (M.F.S.); h.hermanns@amc.uva.nl (H.H.); 2 Department of Anesthesiology, Medical Faculty, Heinrich-Heine-University Düsseldorf, Moorenstrasse 5, Düsseldorf 40225, Germany; robert.werdehausen@med.uni-duesseldorf.de

**Keywords:** anesthetics, local, toxicity, adverse event

## Abstract

This review summarizes current knowledge concerning incidence, risk factors, and mechanisms of perioperative nerve injury, with focus on local anesthetic-induced neurotoxicity. Perioperative nerve injury is a complex phenomenon and can be caused by a number of clinical factors. Anesthetic risk factors for perioperative nerve injury include regional block technique, patient risk factors, and local anesthetic-induced neurotoxicity. Surgery can lead to nerve damage by use of tourniquets or by direct mechanical stress on nerves, such as traction, transection, compression, contusion, ischemia, and stretching. Current literature suggests that the majority of perioperative nerve injuries are unrelated to regional anesthesia. Besides the blockade of sodium channels which is responsible for the anesthetic effect, systemic local anesthetics can have a positive influence on the inflammatory response and the hemostatic system in the perioperative period. However, next to these beneficial effects, local anesthetics exhibit time and dose-dependent toxicity to a variety of tissues, including nerves. There is equivocal experimental evidence that the toxicity varies among local anesthetics. Even though the precise order of events during local anesthetic-induced neurotoxicity is not clear, possible cellular mechanisms have been identified. These include the intrinsic caspase-pathway, PI3K-pathway, and MAPK-pathways. Further research will need to determine whether these pathways are non-specifically activated by local anesthetics, or whether there is a single common precipitating factor.

## 1. Introduction

Peripheral [1] and neuraxial [2,3] regional anesthesia (RA) techniques are widely used worldwide. These methods primarily rely on the injection of local anesthetics (LA) to reversibly block neuronal voltage-gated sodium channels (VGSC) and to thereby reversibly interrupt nerve impulse propagation [4]. Overall, this leads to a reduction in perioperative pain and the systemic effects of surgical stress after certain types of surgery [1,5].

Next to this “classical effect” of local anesthetics, the past decade has seen a new appreciation of other beneficial effects, which are not mediated by blockade of VGSC [6]. Besides the nervous system, local anesthetics have a positive influence on the inflammatory response and the hemostatic system. In particular, G-protein coupled receptors of the G_q/11_ subfamily are involved in anti-inflammatory effects of local anesthetics [7], and the intravenous administration of lidocaine has been shown to duplicate a substantial share of the effects of regional anesthesia after visceral surgery [8].

However, besides these beneficial effects, local anesthetics are toxic to a variety of tissues [9,10] and may contribute to perioperative nerve damage [11]. Local anesthetic induced direct nerve injury can already occur at clinical concentration levels [12,13]. The pathways leading to nerve damage are incompletely understood. Further, the exact incidence of nerve damage due to LA-induced neurotoxicity remains to be determined [1], even as it is recognized that the incidence varies with anesthetic technique, type of surgery, and patient factors [14].

The aim of this review is to summarize current knowledge concerning the incidence, risk factors, and mechanisms of local anesthetic-induced neurotoxicity. Perioperative nerve injury is a complex phenomenon and can be caused by a multitude of clinical factors. While our main focus will be on the neurotoxic effects of local anesthetics, we will also briefly delineate other risk factors and mechanisms that lead to perioperative nerve injury.

The assessment of the incidence of neurotoxicity due to local anesthetics depends on being able to distinguish these different causes of nerve injury. After reviewing the alternate factors, which are divided into anesthetic (mechanical), surgical, and patient factors, we will discuss the mechanisms involved in perioperative nerve injury at a cellular level.

## 2. Methods

In order to summarize and give an overview of the current literature on the incidence, risk factors and mechanisms of nerve damage and neurotoxicity induced by local anesthetics, we collected relevant publications using PubMed and Google Scholar. The search strategy consisted of using MeSH terms “Anesthesia, Conduction”, “Nerve Block”, “Nerve Block/adverse effects”, “Anesthetics, Local”, “Anesthetics, Local/adverse effects”, “Anesthetics, Local/toxicity”, “Anesthetics, Local/complications” and free text words such as regional anesthesia, local anesthetics, adverse effects, neurotoxicity, peripheral/central nerve, injury, mechanism, pathology, etiology, incidence, epidemiology, complications. Articles not mentioning regional anesthesia or nerve injury were excluded.

Thereafter, a critical appraisal was performed on the literature. We reviewed publications for additional relevant information and retrieved articles from the reference lists. Several publications were added by this “snowballing literature” method. The search included articles published until 1 October 2015.

## 3. Literature Review

### 3.1. Local Anesthetics

Local anesthetics have been in clinical use for more than 100 years and drugs such as cocaine, procaine, tetracaine, and lidocaine were introduced with little preclinical testing for possible adverse effects [15]. Even though local anesthetics are a chemically heterogeneous group they all reversibly affect voltage-gated sodium channels (VGSC) in order to inhibit signal conduction along the axon [6]. The target binding site of local anesthetics at the VGSC is located on the intracellular side of the sodium channel, and binding of local anesthetics causes a conformational change which inactivates the channel (the major effect) and creates a positive charge in the lumen of the VGSC, effectively blocking positively charged sodium ions from passing through the channel (the minor effect) [6]. All currently used local anesthetics consist of a lipophilic aromatic group, a hydrophilic amide group, and a linking chain. According to the nature of this chain, local anesthetics can be chemically distinguished into amino-amides, or amino-esters. Whereas the amino-amides are metabolized by the liver’s mixed-function oxidase system, amino-esters are metabolized by blood and tissue esterases [6].

Local anesthetics can enter the nerve cell along three pathways: “classic hydrophilic”, “hydrophobic”, or “alternative hydrophilic” [6]. In the classic hydrophilic pathway accountable for most of the clinical effects of LA, the drug is deposited extracellularly, and exists in equilibrium between the uncharged form and a protonated form. The precise share of uncharged and protonated form is determined by the pKa value and tissue pH. Only the uncharged form can cross the cellular membrane, whereas intracellularly, the drug needs to be conjugated to a hydrogen ion before it can bind to the local anesthetic receptor [6]. There are local anesthetics (e.g., benzocaine) that reach the VGSC directly through the membrane and the lateral fenestrations of the channel; this is characteristic of the hydrophobic pathway [6]. The third or alternative *hydrophilic* pathway is that of permanently charged local anesthetics, such as the lidocaine derivative QX-314. It only very slowly crosses the membrane, due to its charge. However, it is also capable of passing through pores large enough to accommodate the molecule (e.g., transient receptor potential–vanilloid [TRPV]) [6].

Local anesthetics not only target VGSC but can also interact with potassium channels, calcium channels, *N*-methyl-d-aspartate (NMDA) receptors, and G-protein coupled receptors [6].

During peripheral nerve blockade as well as epidural blockade, relatively large amounts of local anesthetics are classically deposited perineurally to generate a concentration gradient of local anesthetic into the nerve [11]. In original descriptions of peripheral nerve blocks, often using landmarks or paresthesia to determine nerve localization, hundreds of mg of local anesthetic were advised. For example, in the case of interscalene block, the recommended dose was 200–250 mg bupivacaine. This enormous dose meant that a substantial share of local anesthetic was absorbed systemically to cause what we now accept as secondary effects, such as anti-inflammatory properties, and the potential mitigation of peri-operative hypercoagulability [6,11].

At present, several local anesthetics with specific properties are clinically available at different concentrations to choose from depending on the clinical preference. For example, they differ in speed of onset, the intensity, and/or the duration of the block. This allows for the tailoring of anesthesia or analgesia to procedure and patient.

### 3.2. Neurological Complications of Regional Anesthesia

Even though regional anesthesia is generally thought to be relatively safe, severe complications periodically occur, and can be devastating for patients [6,16,17]. Systemically, local anesthetic systemic toxicity (LAST) is the most feared acute complication of RA, potentially causing central nervous system toxicity (seizures) and cardiovascular toxicity (arrhythmia, cardiovascular collapse) [6,17].

At the site of injection, nerve damage due to needle injury, hematoma, or local anesthetic toxicity is the most relevant concern. In the early 1990s, cases of cauda equina syndrome (CES) were reported after the introduction of the microcatheter technique for continuous spinal anesthesia with hyperbaric 5% lidocaine and later transient symptoms were described after single dose injections of, among others, spinal lidocaine [18,19,20]. The occurrence of CES heavily suggested that lidocaine had neurotoxic side effects, which were aggravated by the pooling of hyperbaric lidocaine in the caudal dural sac when spinal catheters were placed and repeatedly dosed. This renewed the interest in local anesthetic-induced neurotoxicity and led physicians to more carefully study the incidence and etiology of regional anesthesia-related neuropathy. In due course, attention was also devoted to the transient symptoms associated with lidocaine use for spinal anesthesia, and this led to the description of the Transient neurologic syndrome (TNS), defined as temporary symptoms of radicular pain without motor deficit occurring after recovery from spinal anesthesia, with spontaneous recovery typically within 72 h [15]. In the peripheral nerve system, injury has been reported after regional anesthesia, with the majority of cases resulting in favorable outcome [21,22].

These complications are due to direct nerve injury and can be caused by mechanical injury, ischemia, the toxicity of local anesthetics, or a combination of factors [11,23,24]. There have been several studies that have tried to identify the cellular processes that may contribute to the toxicity of local anesthetics. As of today, multiple pathways have been identified as a possible mechanism for neurotoxicity, however no sole dominant pathway has been found [10,11,23,24]. Models have shown that the toxicity of local anesthetics is time and dose dependent and that toxicity levels differ among local anesthetics [6,25,26]. In order to further reduce the incidence of complications associated with local anesthetics it is not only necessary to reflect on the well-known technical complications of regional anesthesia, but to also have a better understanding of the underlying mechanisms of local anesthetic neurotoxicity.

### 3.3. Incidence of Perioperative Nerve Injury Related to Regional Anesthesia

Regional anesthesia can be divided into central and peripheral nerve blocks.

#### 3.3.1. Central Nerve Blocks

The incidence of clinically relevant nerve damage has been estimated, through prospective and retrospective studies at <4:10.000 for central nerve blocks (CNB) [22]. Neurologic complications that can arise from central nerve blocks include radiculopathy/neuropathy, cauda equina syndrome, intracranial events, and paraplegia. Intracranial events and paraplegia are very rare complications with an incidence of less than 1 in 100.000 for both spinal and epidural anesthesia [22]. Radiculopathy/neuropathy are more common and are estimated to occur 3.78 out of 10.000 spinal anesthetic procedures and 2.19/10.000 for epidural anesthesia [22]. Administration of spinal anesthesia carries a 0.11/10.000 risk of a patient developing cauda equina syndrome, while epidural anesthesia has a somewhat higher risk of 0.23/10.000 [22]. But it should be noted that for the individual patient, this varies according to type of surgery and comorbidities. For example, types of surgery, such as vascular and cardiac surgery, necessitate specific degrees of heparinization, theoretically increasing risk of epidural hematoma. Further, patient comorbidities such as anticoagulant therapy are recognized risk factors for hematoma formation. In all, these more high-risk patients have been estimated to incur serious bleeding complications after epidural placement in 1:4.000 cases, whereas healthy patients, physiologically hypercoagulable, are estimated to suffer from epidural hematoma in only 1:150.000 blocks [14].

#### 3.3.2. Peripheral Nerve Blocks

In peripheral nerve blocks (PNB) the incidence of neurological complications of any severity is considerably higher than for CNB. In the case of PNB, studies estimate the incidence at <3:100. Most of these however are transient sensory deficits, while permanent injury is very rare [16,22,27]. There have been several large prospective studies examining neurologic complication in peripheral nerve blocks [21,22,27,28,29,30] estimating the risk of nerve injury to be between 0.02% and 0.5%. One of these found a potential risk of 0.5%, but after thorough neurologic examination only a minority of these cases could be attributed to the peripheral nerve blocks lowering the risk to 0.04% [27].

The reported incidence however varies considerably between studies and over time, possibly due to methods used to capture anesthesia-related neurologic complications [22,27]. For example, Urban and Urquhart determined that at two weeks postoperatively after receiving a brachial plexus block, 3%–5% of patients suffered from neurological deficits [31]. However, only 0.4% experienced deficits beyond four weeks [31].

### 3.4. Neuroanatomy

Discussion of risk factors for nerve injury necessitates a brief recapitulation of the schematic anatomy of a nerve. The nerve consists of neural tissue surrounded by vasculature and connective tissue. Neurons are embedded in connective tissue and Schwann cells, collectively referred to as endoneurium, which in addition contains capillary blood vessels. Multiple neuronal fibers with the surrounding endoneurium are grouped together in bundles, called fascicles. A protective barrier, known as the perineurium, surrounds each fascicle.

The nerve is built from a collection of fascicles, and is in turn surrounded by the epineurium. An important difference between the epineurium and the perineurium is that the latter is part of the blood-nerve barrier, and hence is capable of protecting neurons from chemical injury [32].

Nerves receive their blood supply in two interconnected ways. The intrinsic blood supply is provided by the capillaries, which are situated in the endoneurium. In addition, there is an extrinsic blood supply that consists of arteries, arterioles, and veins in the epineurium.

### 3.5. Risk Factors for Nerve Injury

There are several mechanisms which contribute to perioperative nerve injury. These can be categorized in anesthetic factors, surgical factors, and patient factors [11,23,24].

The anesthetic factors can be further divided into mechanical or chemical. In this section, we will describe the mechanical risk factors associated with regional anesthesia, while the chemical factors related to the neurotoxicity of local anesthetics will be discussed later.

#### 3.5.1. Anesthetic Factors

In this section, we discuss several technical, anatomical, and equipment factors that influence the risk of peripheral nerve injury due to application of the local anesthetics.

Somewhat surprisingly, it turns out that the incidence of perioperative peripheral nerve injury in exemplary types of orthopedic surgery does not depend on whether regional or general anesthesia is applied. Studies show that epidural anesthesia and general anesthesia are associated with perioperative nerve injury (PNI), but that peripheral nerve block is not an independent risk factor [33]. Similarly, it is found that PNB is not associated with PNI following reconstructive knee, hip or shoulder surgery [34,35,36]. This suggests that the large majority of nerve injuries are due to causes other than the nerve block.

The incidence of peripheral nerve injection injury with local anesthetic is dependent among others on the location of the injection [1,22]. For example, sequelae in closed claims analyses are most frequently reported for brachial plexus blocks but it is not clear whether this is connected to the fact that brachial plexus blocks are also performed very frequently [1,22]. The same differential incidence can also be observed at a smaller anatomical level, as the chance of nerve injury is thought to be highest with intrafascicular application of the anesthetics, and lower if local anesthetic is deposited extraneurally [23]. Although peripheral nerves are largely composed of connective tissue, direct trauma with needle or catheter can still result direct nerve perforation, and injury to fascicle and/or perineurium. When mechanical damage occurs to the perineurium, the environment contained within the perineurium loses its protection. This theoretically increases the chance of further nerve injury [11].

The size and type of needle used for the injections also affect the chance of nerve injury. Although long-beveled needles have a higher chance of causing nerve punctures, the injuries that occur due to short-beveled needles are generally more severe [37].

Not only does intrafascicular application of local anesthetics pose a risk of mechanically damaging nerves, it also exposes the nerves to much higher concentrations of local anesthetics and hence it increases the associated neurotoxic effects [25,26,38].

Another cause of peripheral nerve injuries is the injection pressure associated with the application of the local anesthetic. The opening injection pressures are higher for intrafascicular injections compared with perineural injections, which is thought to translate into high pressure in the injected compartment. The latter has been associated with a higher risk of nerve injury in experimental studies [39].

Theoretically, ultrasound, which is now widely used for regional anesthesia, should lead to a reduction of needle-nerve contacts and mechanical trauma as opposed to nerve stimulator- or paresthesia-based approaches. Indeed, a higher success rate and a reduction of local anesthetic volume needed for adequate blockade have been described [6]. However, in practice the ability to visualize both the needle tip and relevant nerves at potential risk seems dependent on the clinical situation and user skills. Indeed, while ultrasound guidance has been effective in reducing the incidence of systemic toxicity, there is no evidence that it decreases the risk of nerve damage.

#### 3.5.2. Surgical Factors

Surgery can stress anatomical structures and can mechanically induce traction, transection, compression, contusion, ischemia, and stretch nerves [40]. The loss of muscle tone during anesthesia also deprives nerves of some of their protection against mechanical forces and makes them more susceptible to injury [23].

The incidence of transient neurologic symptoms varies between procedures, most likely because of factors such as patient positioning and the use of tourniquets [11,23,24,40,41]. For example, patients receiving spinal anesthesia in the lithotomy position have a higher risk of developing TNS then patients undergoing surgery in the supine position [24].

Tourniquets by design inflate to high pressures and are capable of damaging nerves by mechanical compression and/or ischemia [11,41]. Large nerves are predominantly affected and high pressures can result in time-dependent motor loss and diminished sensory perception [11,41].

Nerve injuries related to surgery have been reported with varying degree of severity. Nerve damage due to surgery may result from physical pressure resulting in ischemia, mechanical stretching, or direct injury [11,40].

#### 3.5.3. Patient Factors

Pre-existing neuropathies and risk factors can compromise the functional integrity of peripheral nerves and make them more susceptible to injury. The potential sources of impaired nerve function include entrapment, metabolic, ischemic, toxic, hereditary [23] and demyelinating diseases [23]. Peripheral vascular disease, vasculitis, cigarette smoking, and hypertension are all medical conditions that affect the microvasculature and therefore may make nerves more vulnerable to ischemic insults induced during the perioperative period [23] but the clinical relevance remains unclear. Preexisting neurological diseases such as multiple sclerosis, Guillain-Barre syndrome, Post-polio syndrome, and diabetic peripheral neuropathy (DPN) may also place nerves at increased risk for injury [42]. DPN in particular is a possible risk factor as diabetes stresses nerves metabolically and hemodynamically [42,43].

As indications for regional anesthesia, especially peripheral nerve blocks continue to expand in some areas, also with the increased use of ultrasound, more patients will be exposed to the possible risk of neurologic complications [44]. Also, the expansion of patient selection will not only increase the use of RA, it will also add patient groups with substantial comorbidities [42]. This opens the possibility that the number of patients confronted with neurologic complications related to RA could increase.

### 3.6. General Aspects of Local Anesthetic-Induced Neurotoxicity

Chemical nerve injury is caused by the toxicity of the applied solution or its additives. *In vitro* models have shown that all local anesthetics exhibit neurotoxic effects, even though there may be variations in neurotoxicity between different local anesthetics. The degree of neurotoxicity of local anesthetic has been demonstrated to be concentration (or dose)-dependent [26]. The adverse effects also increase with the duration of the exposure. In clinical practice, the doses of local anesthetics used has generally decreased over time, and the high concentrations of lidocaine (5%) conjectured with the occurrence of cauda equina syndrome are not available any more, the highest concentration routinely used for nerve block nowadays being 2%. Likewise, bupivacaine 0.75% has been abandoned in Europe, and the highest concentration used now is 0.5%. There is experimental evidence that the toxicity among local anesthetics varies. These works suggest that lidocaine is more toxic than equipotent concentrations of bupivacaine [45,46]. However, this is still up for debate as other studies have shown that there is no difference in toxicity between local anesthetics [26,47].

Local anesthetics have local vasoconstrictive properties and can theoretically damage nerves through ischemia, although the relevance of this mechanism for currently used local anesthetics is unclear [11]. The adjuvant epinephrine is often used to increase block duration through vasoconstriction. However, this extends the exposure of nerves to local anesthetics and further decreases blood flow, putting nerves at greater risk of ischemic damage [11]. The oxidative injury resulting from ischemia and reperfusion leads, in addition to neuronal damage, to the initiation of apoptosis by affecting the Schwann cells. In clinical practice, the use of lower concentrations of short-acting local anesthetics (e.g., lidocaine 1%–2% and mepivacaine 1.5%) combined with adrenaline 1:200.000–1:100.000 is considered safe.

### 3.7. Cellular Mechanisms of Local Anesthetic-Induced Neurotoxicity

**Figure 1:**
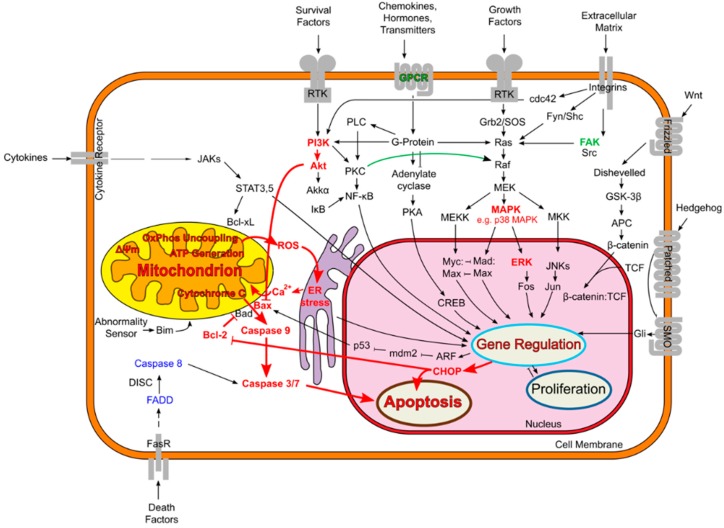
Schematic illustration of relevant intracellular signal transduction pathways of local anesthetic-induced toxicity. Signaling pathways and mediators that are conveying a toxicity-increasing effect are marked in red color, while those that are involved in protective mechanisms are highlighted green. Blue colored elements were proven not to be affected, while the role of non-highlighted (black) elements remains unclear or not investigated. Local anesthetic neurotoxicity can affect multiple signaling pathways and ultimately leads to the activations of mitochondrial signaling pathway of apoptosis, which is also referred to as the intrinsic pathway of apoptosis. This pathway involves mitochondrial injury and dysfunction, loss of mitochondrial membrane potential (ΔΨ_m_), and the oligomerization of Bax protein in the outer mitochondrial membrane, which leads to the release of apoptosis-inducing mediators like cytochrome c. This leads to the formation of the apoptosome, which comprises caspase-9 and other factors. The apoptosome, in turn, activates the effector caspases 3 and 7 and therefore converges into a signaling cascade, which ultimately leads to specific nuclear DNA-fragmentation. Abbreviations: adenomatous polyposis coli tumor-suppressor gene (APC); alternate reading frame tumor suppressor protein (ARF); CCAAT-enhancer-binding protein homologous protein (CHOP); endoplasmatic reticulum (ER); extracellular-signal-regulated kinase (ERK); Fas-associated protein with Death Domain (FADD); focal adhesion kinase (FAK); mitogen-activated protein kinase (MAPK); reactive oxygen species (ROS); Smoothened protein (Smo) T-cell factor (TCF). This figure was prepared based on a previous figure by Hanahan and Weinberg [63] and a previously modified version (http://en.wikipedia.org/wiki/File:Signal_transduction_v1.png).

All local anesthetics are neurotoxic in a dose-dependent manner [26]. However, it should be noted that the exact pathway of cell death seems to depend on the concentration of local anesthetic. In Jurkat cells (immortalized lymphocytes), for example, clinically relevant concentrations of lidocaine induced apoptosis, whereas higher concentrations caused unspecific cell death and necrosis [48,49]. There are other possible mechanisms that bring about apoptosis in neurons. Local anesthetics can lead to fragmentation of DNA and disrupt the membrane potential in mitochondria. This results in the uncoupling of the oxidative phosphorylation, which subsequently causes the release of cytochrome c and the initiation of the caspase pathway, possibly leading to apoptosis. Examples of other pro-apoptotic enzymes that can be activated by local anesthetics are p38 mitogen-activated protein kinase and Jun N-terminal kinase. The cellular mechanisms responsible for the neurotoxic effects of local anesthetics will be discussed in more detail below. Even if detailed subcellular pathways of toxicity have been the subject of substantial research efforts, the “big picture” is still unclear. In the following sections, some important subcellular pathways and their potential relevance for local anesthetic-induced neurotoxicity are presented. See Figure 1.

#### 3.7.1. Sodium Channel Blockade

The classical primary target of local anesthetics is the sodium channel, but this mechanism is regarded as unrelated to the neurotoxic effects of lidocaine. This can be inferred from studies showing that extracellular blockade of the sodium channel by, e.g., tetrodotoxin (TTX) does not lead to neurotoxicity [49].

#### 3.7.2. G-protein Coupled Receptors

Over the past decade, ample research efforts have addressed the alternative mechanisms of action of local anesthetics mediated by G-protein coupled receptors (GPCR). [50] These mechanisms account for the systemic anti-inflammatory effects of LAs, mediated by G-proteins of the G_q/11_ family, which predominantly influence hemostatic and inflammatory signaling. Examples include the lysophosphatidic acid (LPA)-, thromboxane (TXA) 2- or platelet activating factor (PAF)-receptors [51].

No investigation to date has reported on neurotoxic effects at the concentrations attained systemically, and no investigation has conjectured GPCR in LA-induced neurotoxicity at higher concentrations such as achieved after local administration.

#### 3.7.3. Caspase Pathways

The complex interplay between survival and death factors is crucial to cell growth and apoptosis [52]. The most central group of molecules in apoptosis is the caspase family. The latter can be activated along the extrinsic or intrinsic pathway. The *extrinsic pathway* is initiated subsequent to Fas- or TNF-receptor activation by their respective ligand, and the combination of Fas-associated death domain with the procaspase-8 to form the death-inducing signaling complex (DISC) [53]. This in turn has two effects: activation of caspase-3 in virtually all cells [52], and cleaving of Bid to tBid, which stimulates the release of Cytochrome c from mitochondria in some cells such as hepatocytes [54]. Cytochrome c release will lead to the creation of the so-called “apoptosome” with the help of caspase-3 [55]. The *intrinsic pathway*, in contrast, is activated by the release of Cytochrome c and other pro-apoptotic factors into the cytosol. These pro-apoptotic effects, especially the release of Cytochrome C, can be counteracted by activation of bcl family molecules [56].

Werdehausen *et al.* investigated the relevance of the death and survival pathways for local anesthetic-induced neurotoxicity [48]. In a Jurkat cell line, lidocaine-induced cytochrome c release and apoptosis via the intrinsic pathway was demonstrated, and overexpression of bcl-2 or deficiency in caspase-9 strongly inhibited apoptosis. Release of cytochrome c into the cytoplasma was also confirmed by Lirk and colleagues when incubating primary sensory neurons with the experimental local anesthetic amitriptyline; they also found that inhibition of caspase-3 attenuated apoptosis [57].

Other studies have described increases in caspase-3 as an event during local anesthetic-induced neurotoxicity [58,59,60,61], and Johnson and colleagues confirmed increased pan-caspase staining after lidocaine incubation in ND-7 cells [49].

On the survival factor side, nerve growth factor (NGF) has been reported to protect against local anesthetic-induced neurotoxicity in an *in vivo* spinal injection model in rats [62].

#### 3.7.4. PI3-Akt Pathway

The phosphoinositide 3-kinase (PI3K) family of enzymes is key in the regulation of cell growth and metabolism and dysregulation has been reported in a wide range of malignancies [64]. PI3K activates serine-threonine protein kinase B (also known as Akt), an enzyme protective against apoptosis under conditions as diverse as glutamate toxicity and oxygen and glucose deprivation [65,66,67].

The relevance of the PI3K pathway for local anesthetic-induced neurotoxicity was underlined by a series of experiments. The first manuscript, published by Ma and colleagues in 2010 [67], investigated the potential neuroprotective effects of dexamethasone in an *in vitro* model of neuroblastoma cells. Dexamethasone was found to increase levels of Akt, and led to attenuated neurotoxicity of bupivacaine and lidocaine. Notably, pharmacologic inhibition of Akt abolished this protective effect of dexamethasone [67]. In a follow-up study, lithium was investigated, again in a model of bupivacaine-induced toxicity on neuroblastoma cells. The authors found that lithium significantly attenuated bupivacaine neurotoxicity, but when Akt was inhibited, this protective effect was lost [68]. Interestingly, the authors also investigated the extracellular signal-regulated kinase (ERK) pathway, the “protective part” of the Mitogen Activated Protein Kinase (MAPK) family, and found that activation of ERK was also key in the protective action of lithium [68]. A further study also identified alpha-lipoic acid as activator of Akt and protector against bupivacaine-induced neurotoxicity [69]. This cross-link to MAPK is interesting, because they have also been implicated in the neurotoxicity induced by local anesthetics.

#### 3.7.5. Mitogen-Activated Protein Kinase (MAPK) Pathway

P38 MAP Kinase activation can be initiated by a variety of environmental stressors, such as radical oxygen species, heat shock, and cytotoxic drugs. In neurons, its effector molecules (e.g., lipoxygenase) influence vital cellular processes such as cellular differentiation, neuronal plasticity, and apoptosis. Inhibition of p38 MAP Kinase activity has been shown to be of potential therapeutic benefit in experimental nerve trauma [70], excitotoxicity [71], and metabolic injury [72]. It was demonstrated *in vitro* and *in vivo* that interference with MAPK pathways may substantially reduce the incidence and extent of local anesthetic-induced neurotoxicity [73]. In particular, the p38 Mitogen Activated Protein (MAP) Kinase (=MAP Kinase 14) is activated in cell cultures incubated with local anesthetics [73]. Currently, both oral and parenteral formulations of p38 MAP Kinase inhibitors are undergoing clinical testing and therefore these drugs may become clinically available [74]. The relevance of the ERK family in mediating protective effects of lithium via the Akt pathway was discussed above [69].

## 4. Discussion

In this review, we summarized current knowledge concerning the incidence, risk factors, and mechanisms of local anesthetic-induced neurotoxicity. Perioperative neurologic complications associated with the use of regional anesthesia are complex and often multifactorial, including surgical, patient, and anesthetic factors, which often complicates finding the exact cause of nerve injury. By combining and summarizing the current literature on these separate factors, we hope to have contributed to a better understanding of regional anesthesia associated nerve injury. The determination of the exact incidence of nerve damage caused by regional anesthesia has been hampered by the heterogeneity of the clinical studies. Over the years, clinical studies have often applied different criteria to determine the frequency of nerve injury. For instance, results from studies in the surgical literature generally indicate a higher incidence of block related nerve injuries than those reported in anesthetic literature. Another complicating factor in discerning the incidence of local anesthetic-induced nerve injury is the possibility of other causes.

The alternate causes of nerve injury other than the neurotoxicity of local anesthetics are mechanical. In addition, there are surgical and patient related risk factors. Many reported cases in literature cite combinations of several factors as responsible for nerve injury. For instance, patients with subclinical pre-existing neurologic conditions may develop symptomatic neuropathies in the post-operative period. The application of one or more stressors on a dysfunctional but clinically normal nerve can result in new neurologic symptoms. Upton and McComas have called this phenomenon the ”double crush” syndrome [75]. Therefore, it may not always possible to determine a single cause to perioperative nerve injuries. A good clinical example to illustrate the multifactorial origin of neuronal damage is perioperative ulnar neuropathy. A combination of different factors such as predisposing conditions (e.g., male sex, over- or underweight, pre-existing asymptomatic ulnar nerve dysfunction, prolonged hospitalization, *etc.*) and perioperative patient positioning leading to nerve injury due to stretching, compression, ischemia, and metabolic derangement have been identified as risk factors [76,77].

Neurotoxicity of local anesthetics is thought to contribute to perioperative neurologic complications. It is therefore important to identify and understand the cellular mechanisms behind these neurotoxic effects. *In vitro* models have shown that the neurotoxicity of local anesthetics is triggered by the effect on the intrinsic caspase-pathway, PI3k-pathway, and MAPK-pathways and not by interaction with VGSC and G-coupled protein receptors [48,67,73]. Particularly interesting are the intricate connections that have been found between the P13k- and MAPK-pathways [69].

It should, however, be kept in mind that translation from preclinical *in vitro* studies to the clinical situation needs to be considered with precaution, given that the predominant share of the experimental studies available have been performed using cell cultures and thus focused on cell bodies and not on axons. Taking this into account, it is not entirely clear to what extent apoptotic and other cellular mechanisms seen in *in vitro* studies effectively contribute to clinical manifestation of LA neurotoxicity.

Given the low incidence of LA induced neurotoxicity, it is intriguing to speculate on protective mechanisms that counteract neurotoxicity elicited by local anesthetics, with respect to discrepancies between different neuronal cell types such as motor and sensory nerve fibers. The experimental data on this is sparse. The only study investigating the differential neurotoxic effect of LA on different subgroups of sensory neurons was performed by Haller *et al.* [73]. Here, the authors could show, in an *in vitro* setting that lidocaine-induced neurotoxicity was identically observed in all different investigated dorsal root ganglion (DRG) cell types. It is unknown whether the fact that clinically, sensory deficits occur more often than impairment of motor function is due to differences in morphology (e.g., size of the nerve fibers, extent of myelination, *etc.*). These morphologic characteristics may provide protection to some degree and beyond that, Schwann cell toxicity may play a substantial pathogenic role [25,78]. Together, these issues might represent interesting research objectives for future investigations.

## 5. Conclusions

Perioperative neurologic complications associated with regional anesthesia are complex and often multifactorial, including surgical, patient, and anesthetic factors. This often makes it very difficult to pinpoint the etiology in clinical cases of perioperative nerve injury. Although clinically relevant nerve damage due to regional anesthesia occurs, it is very uncommon for it to become permanent. At the cellular level, the neurotoxicity of local anesthetics is caused by the effect on the intrinsic caspase-pathway, PI3k-pathway, and MAPK-pathways. Current literature suggests that the majority of perioperative nerve injuries are unrelated to regional anesthesia.
